# Preparation of Monoclonal Antibodies Specifically Reacting with the Trichothecene Mycotoxins Nivalenol and 15-Acetylnivalenol via the Introduction of a Linker Molecule into Its C-15 Position

**DOI:** 10.3390/toxins14110747

**Published:** 2022-10-31

**Authors:** Kyoko Noda, Yuki Hirakawa, Tomomi Nishino, Ritsuto Sekizuka, Marin Kishimoto, Tomohiro Furukawa, Sakiko Sawane, Ayu Matsunaga, Naoki Kobayashi, Kazutoshi Sugita, Kenji Oonaka, Hiroko Kawakami, Yuji Otsuka, Tetsuya Yamamoto, Toshihiro Yamamoto, Taku Yoshiya, Maiko Watanabe, Machiko Saka, Keiko Momma, Masayo Kushiro, Shiro Miyake

**Affiliations:** 1 Department of Food and Life Science, Azabu University, Sagamihara 252-5201, Japan; 2 Department of Nutrition and Food Science, Ochanomizu University, Bunkyo 112-8610, Japan; 3 Department of Food and Nutrition, Kyoto Women’s University, Kyoto 605-8501, Japan; 4 National Agriculture and Food Research Organization, Tsukuba 305-8642, Japan; 5 Department of Veterinary Medicine, Azabu University, Sagamihara 252-5201, Japan; 6 Peptide Institute, Inc., Osaka 567-0085, Japan; 7 National Institute of Health Sciences, Kawasaki 210-9501, Japan; 8 Eco-Science Co., Nagano 381-0006, Japan

**Keywords:** ELISA, immunoassay, *Fusarium* head blight, nivalenol, hapten

## Abstract

Nivalenol (NIV) is a trichothecene mycotoxin that is more toxic than deoxynivalenol. It accumulates in grains due to infection with *Fusarium* species, which are the causative agents of scab or *Fusarium* head blight. An immunoassay, which is a rapid and easy analytical method, is necessary for monitoring NIV in grains. However, a specific antibody against NIV has not been prepared previously. To establish an immunoassay, we prepared NIV, introduced a linker, and generated antibodies against it. NIV was prepared from a culture of *Fusarium kyushuense* obtained from pressed barley through chromatographic procedures with synthetic adsorbents and silica gel. NIV was reacted with glutaric anhydride, and the reaction was stopped before mono-hemiglutaryl-NIV was changed to di-hemiglutaryl-NIV. 15-*O*-Hemiglutaryl-NIV was isolated via preparative HPLC and bound to keyhole limpet hemocyanin (KLH) using the active ester method. Two different monoclonal antibodies were prepared by immunizing mice with the NIV-KLH conjugate. The 50% inhibitory concentration values were 36 and 37 ng/mL. These antibodies also showed high reactivity in a direct competitive enzyme-linked immunosorbent assay and specifically reacted with NIV and 15-acetyl-NIV but not with deoxynivalenol and 4-acetyl-NIV.

## 1. Introduction

**Figure 1:**
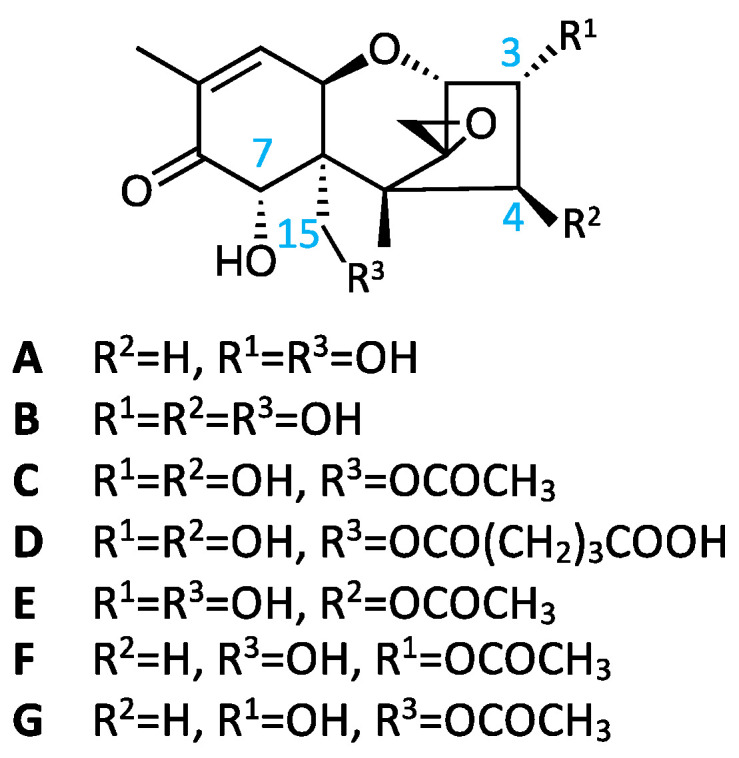
Chemical structures of deoxynivalenol (DON), nivalenol (NIV), and relative compounds. **A**, DON; **B**, NIV; **C**, 15-acetylnivalenol (15-Ac-NIV); **D**, 15-*O*-hemiglutarylnivalenol (15-Glt-NIV); **E**, 4-acetylnivalenol (fusarenon X or 4-Ac-NIV); **F**, 3-acetyldeoxynivalenol (3-Ac-DON); **G**, 15-acetyldeoxynivalenol (15-Ac-DON).

Worldwide, more than 700 million tons of wheat is produced annually, which feeds two-thirds of the population, along with rice and corn [1]. The toxicological and nutritional aspects of wheat are particularly important for human consumption [2]. Humid and temperate weather during wheat anthesis often results in scab or *Fusarium* head blight, a disease caused by fungi of several *Fusarium* species. This disease causes two forms of agricultural damage, specifically a threat to food security caused by low harvest quality and quantity and a threat to food safety owing to mycotoxin accumulation in grains. Among these mycotoxins, the trichothecene mycotoxin deoxynivalenol (DON; **A** in Figure 1) is the most hazardous to wheat worldwide [3]. In addition to DON, another trichothecene mycotoxin, nivalenol (NIV; **B** in Figure 1), represents a potential risk because of its frequent co-occurrence with DON [4]. NIV has been reported to be more toxic to animals than DON [5]. Using the lowest observed adverse effect level of 0.7 mg/kg body weight per day with a safety factor of 1000, the European Commission established a temporary tolerable daily intake level of 0.7 μg/kg body weight for NIV, whereas this value was reported to be 1.0 μg/kg body weight for DON [6]. Further, a higher occurrence of NIV than DON has been reported worldwide [7]. The natural occurrence of 15-acetylnivalenol (15-Ac-NIV; **C** in Figure 1) was reported in Norwegian strains of *Fusairum equiseti* in 2001 [8]. In that report, the cytotoxicity of *F. equiseti* in vitro (rice culture) was mainly attributed to 15-Ac-NIV with fusarochromanone. However, no reports of 15-Ac-NIV have been published since then, and it is assumed that its contamination frequency and concentration are low.

A rapid and easy analysis method is necessary for monitoring NIV in wheat and other grains. NIV analysis is generally performed using high-performance liquid chromatography equipped with a UV detector (HPLC-UVD) or tandem mass spectrometry (LC-MS/MS) [9,10,11,12]. These methods are sensitive and accurate, but not suitable for rapid and easy testing in the agricultural field because of time constraints and complex sample preparation techniques; moreover, these instruments cannot be moved from the laboratory to the field. As an alternative method, we have focused on immunoassays, which are based on the specific reactions between antigens and antibodies. Immunoassays are often used to monitor mycotoxins in agricultural products [13,14,15]. These are simple, rapid, and cost-effective methods compared to HPLC-UVD or LC-MS/MS, but it is essential to prepare antibodies that react with the measurement target for their application. Antibodies specifically reacting with DON have been reported in various studies [16,17,18,19,20,21,22]. However, no reports of antibodies specifically reacting with NIV were found in previous studies, except for that by Maragos et al., who prepared a monoclonal antibody (MoAb) that showed 57.4% cross-reactivity with NIV when compared to reactivity with DON [23].

Because NIV and DON are haptens, they must be covalently bound to proteins, such as keyhole limpet hemocyanin (KLH), through an adequate linker, for antibody preparation via immunization. As shown in Figure 1, DON does not have an OH group at the 4-position of the carbon atom in its chemical structure, whereas NIV does [24,25]. Hence, NIV contains four OH groups in its structure. These OH groups can react equally with the linker, and their introduction into several positions distorts the original chemical structure. This might explain why it is difficult to prepare antibodies that react with NIV. The introduction of a linker specifically into one of the OH groups in NIV was therefore examined. In this study, the designed hapten 15-*O*-hemiglutaryl-NIV (15-Glt-NIV; **D** in Figure 1), the preparation of polyclonal antibodies (PoAbs) and MoAbs, and the reactivity of these antibodies with NIV via a direct competitive enzyme-linked immunosorbent assay (dc-ELISA), which is a representative immunoassay for the determination of mycotoxins, are described.

## 2. Results and Discussion

### 2.1. Preparation of NIV

The preparation of 200 mg of NIV was necessary for the synthesis of 15-Glt-NIV and 15-Ac-NIV. Although a purification method using centrifugal partition chromatography was reported by Onji et al. [26], we examined the preparation of NIV from the culture medium using a column chromatographic procedure with synthetic adsorbents and silica gel without an expensive instrument. According to previous papers, NIV is generally extracted with acetonitrile [9,10,11,12,26,27]. However, large amounts of lipid and brown hydrophobic substances produced by *Fusarium kyushuense* used in this study were also found to be extracted simultaneously with acetonitrile, which made it difficult to separate NIV from the extract. These impurities were not extracted on using water. NIV is easily extracted with water because it has four hydroxy groups. Considering the difference in solubility between NIV and the impurities, water was used as the solvent. After the extracts containing NIV were adsorbed to the DIAION HP20 resin, the removal of brown hydrophilic impurities that were co-extracted with water was drastically improved by washing the resin with water, compared to that with conventional acetonitrile extracts. The combination of extraction with water and chromatography with synthetic adsorbents made it easy to remove impurities from the extract and concentrate the eluate containing NIV with methanol. However, it was difficult to dissolve the concentrated eluate from the resin in the ethyl acetate/methanol (8:1, *v/v*) solvent for silica gel chromatography. The paste containing NIV was dispersed through ultrasonication and adsorbed to 1 mL of silica gel. After purification via silica gel column chromatography, the crude NIV was dissolved in 5% (*v/v*) acetonitrile in water. After it was maintained at 4 °C, high-purity NIV was saturated and precipitated as a white powder, showing a single peak with HPLC-UVD (Figure S1). As a result, 200 mg of NIV was obtained from 1800 g of pressed barley culture of *Fusarium*.

### 2.2. Introduction of a Carboxy Group into NIV

Although specific antibodies targeting DON were generated through the extension of a linker from the OH groups of DON in previous studies, the preparation of specific antibodies against NIV had not been reported, except for that by Maragos et al. They successfully prepared monoclonal antibodies with 57.4% cross-reactivity with NIV, as compared to reactivity with DON [23]. They devised a method to modify NIV through *O*-methylglycine binding and isolated the mono-substituted derivatives after removing the methyl ester to prepare the aforementioned MoAb. This was determined to be useful in assessing both DON and NIV, but it could not be used to detect NIV alone. To prepare NIV-specific antibodies without cross-reaction with DON, an extension between NIV and the protein using glutaric anhydride as a linker and the selective introduction of a linker molecule into one of the OH groups of NIV were examined.

NIV has four OH groups at the C-3, 4, 7, and 15 positions in its structure, whereas DON has three OH groups at the C-3, 7, and 15 positions, as shown in Figure 1-**A** and Figure 1-**B**. The presence or absence of an OH group at the C-4 position represents the difference between NIV and DON. However, why this difference affects antibody production remained unclear. It has been reported that the OH groups at the C-7 positions of NIV and DON are unreactive [23,28,29], which could be due to steric hindrance mediated by the hydroxymethyl group at the C-15 position. We understood that this was the reason as to why the OH groups at the C-3 and C-15 positions were the link-introducing sites among the three OH groups in DON. When the linker molecule was introduced into DON at an adequate ratio without a protecting group, the C-3 or C-15 position would be predominantly used for the binding site. Accordingly, antibodies showing high reactivity with DON were prepared. However, it would have been difficult to introduce a linker predominantly into one OH group among the three reactive OH groups in NIV without using protective groups, as well as to attach protective groups selectively to two OH groups.

**Figure 2:**
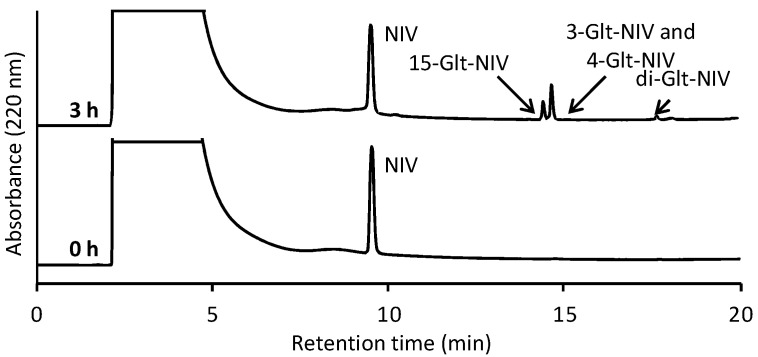
Representative chromatograms from the reaction between nivalenol (NIV) and glutaric anhydride based on high-performance liquid chromatography equipped with a UV detector. The bottom shows the reaction solution at 0 h and the top shows it at 3 h.

Therefore, the selective introduction of a linker molecule into one of the OH groups of NIV was examined. The OH group at the C-15 position in NIV was coupled with a primary carbon atom. This OH group might be slightly different from others coupled with a secondary carbon atom in terms of the reactivity and/or chemical properties of the ester derivatives. NIV was reacted with a three-fold greater amount of glutaric anhydride for 3 h at 25 °C. Figure 2 shows a typical profile of the reaction mixture using reversed-phase HPLC equipped with a UV detector. After this HPLC-UVD analysis, this reaction solution was also analyzed by LC-MS, to confirm molecular weight of products. Two peaks with the same molecular weight as mono-Glt-NIV, *m/z* 449.1 ([M + Na]^+^) and one peak with the same molecular weight as di-Glt-NIV, *m/z* 563.1 ([M + Na]^+^) were observed (Figures S2–S4). Comparing the peak areas, 71% of NIV remained unreacted under these reaction conditions. Although only two peaks derived from mono-Glt-NIV were observed, it seemed that three types of mono-Glt-NIV were formed at an yield of 27%. Di-Glt-NIV was formed at an yield of 2%. It was expected that more di-Glt-NIV would be formed via mono-Glt-NIV based on a longer reaction, making it difficult to isolate mono-Glt-NIV. The reaction was stopped after 3 h, and the products were purified and identified.

^1^H-NMR data of the compound purified from the peak at a retention time of approximately 14.4 min showed that this purified compound was a type of mono-Glt-NIV (Figure S5). The chemical shift of protons at the C-15 position was downfield from NIV (δ = 4.42 and 4.07), whereas those at the C-3 and C-4 positions were not. These data show that the product was 15-Glt-NIV. However, the ^1^H-NMR data of the products purified from the peak at a retention time of approximately 14.7 min showed that these products were a mixture of two types of NIV derivatives with a hemiglutaryl group, namely 3-Glt-NIV and 4-Glt-NIV, and 3-Glt-NIV was formed more readily than 4-Glt-NIV (Figure S6). However, these derivatives could not be separated. ^1^H-NMR data of di-Glt-NIV purified from the peak at a retention time of approximately 18.0 min showed that this compound was 3,15-di-Glt-NIV (Figure S7). Because 4-Glt-NIV was barely formed, it seemed that di-Glt-NIV with a glutaryl group at the C-4 position was not readily formed. Further, no difference in reactivity was observed among the three OH groups of NIV under this reaction condition. Instead, 15-Glt-NIV was separated from mono-Glt-NIV via reversed-phase chromatography. Thus, we repeated the isolation of the peak at a retention time of approximately 14.4 min and succeeded in preparing a derivative of NIV with one hemiglutaryl group, namely 15-Glt-NIV.

### 2.3. Preparation of PoAbs That React with NIV

**Figure 3:**
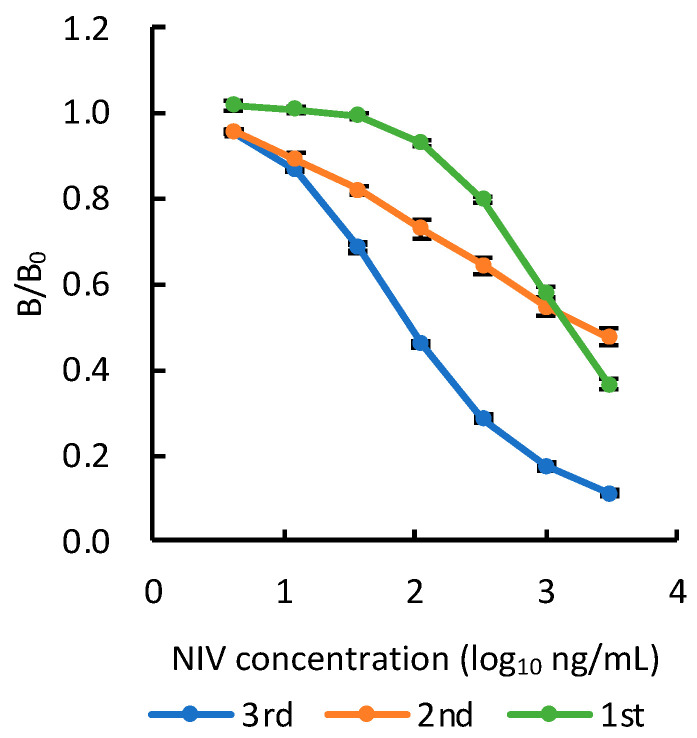
Reactivity of polyclonal antibodies (PoAbs) after each booster immunization with nivalenol (NIV), as determined via a direct competitive enzyme-linked immunosorbent assay (dc-ELISA). Green, orange, and blue lines show the mean values for reaction of the PoAbs from three mice after the 1st, 2nd, and 3rd booster immunizations, respectively. B/B_0_ = ratio of absorbance with NIV to absorbance without NIV. Each data point is the mean of three replicates based on independent examinations; error bars indicate ± SD.

The reactivity of antibodies against haptens greatly depends on the structure of the hapten bound to proteins [30,31], but also sometimes depends on the immunization schedule. An adequate schedule for the NIV-KLH conjugate, for which the titer generally reaches a plateau with two booster immunizations [32,33], was confirmed to produce a highly reactive antibody against NIV using PoAbs from immunized mice. As shown in Figure 3, after the 1st booster immunization, the PoAb reacted weakly with NIV at the 50% inhibitory concentration (IC_50_) of 1400 ng/mL. The slope became less severe after the 2nd booster immunization, whereas the PoAb reacted with NIV in a range of less than 100 ng/mL, a range at which the PoAb did not react after the 1st booster immunization. This was considered the reason for the increase in the amount of antibody that reacted with NIV at a lower concentration range, maintaining the amount of antibody that reacted with high concentrations of NIV. After the 3rd booster immunization, the PoAb showed high reactivity with NIV at an IC_50_ value of 90 ng/mL, with a steep inhibition curve slope. Moreover, the antibody that reacted with NIV in a higher concentration range disappeared. Similar results were obtained for all three mice. Thus, three rounds of booster immunizations were useful for the preparation of highly reactive antibodies. The prepared PoAb showed similarly high reactivity with the target NIV compared to that of the other haptens, as described previously [32,33]. The reasons for this result are uncertain and they can be multiple. The hapten structure, carrier protein, conjugate structure, immunization procedure could account for this result. Changes in reactivity were presumably caused by a class change in antibodies and the enhancement of affinity via repeated immunization with the NIV-KLH conjugate, although the reason for affinity improvements against NIV-KLH being delayed compared to that with the usual immunogens was not clear.

### 2.4. Preparation of MoAbs That React with NIV

The immunized mice were used for the preparation of MoAbs. After cell fusion and incubation at 37 °C for 7–10 days, the MoAbs secreted from the colonies of hybridoma cells were screened using direct ELISA (d-ELISA) based on their reactivity with horseradish peroxidase (HRP)-labeled NIV. Since the immunogen was an NIV-KLH conjugate, MoAbs that reacted with the common structure between NIV-KLH and HRP-labeled NIV were screened. The MoAbs were further screened based on their reactivity with NIV using dc-ELISA. The established cells secreting the anti-NIV MoAbs included two clones, MNV80 (IgG_1_, κ) and MNV87 (IgG_1_, κ). The supernatants of the cultured media were used as the MoAb solutions. The MoAbs were respectively named MNV80 and MNV87, as with these cells.

### 2.5. Reactivity of PoAbs and MoAbs with NIV Based on dc-ELISA

**Figure 4:**
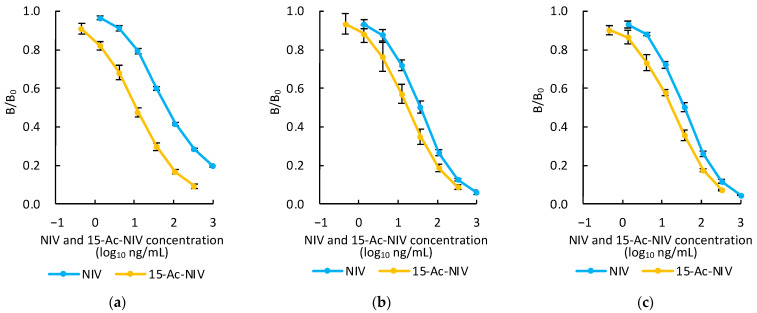
Reactivity of polyclonal antibodies (PoAbs; after the 3rd booster immunization) (**a**) and monoclonal antibodies (MoAbs) MNV80 (**b**) and MNV87 (**c**) with nivalenol (NIV; blue line) and 15-acetyl-NIV (15-Ac-NIV; orange line) based on a direct competitive enzyme-linked immunosorbent assay. B/B_0_ = ratio of absorbance with NIV or 15-Ac-NIV to absorbance without NIV. Each data point is the mean of five replicates for NIV and three replicates for 15-Ac-NIV based on independent examinations; error bars indicate ± SD.

The PoAbs after three rounds of booster immunizations and the MoAbs, MNV80 and MNV87, were used to confirm reactivity with NIV via dc-ELISA. Figure 4 shows that the PoAbs, MNV80, and MNV87 reacted with NIV in the ranges of 12–950 ng/mL, 6.9–180 ng/mL, and 7.1–170 ng/mL, respectively. These ranges were defined as concentrations between the IC_20_ and IC_80_ values. The IC_50_ values of PoAbs, MNV80, and MNV87 were 70, 36, and 37 ng/mL, respectively. The maximum permitted level of DON in flour, meal, semolina, and flakes derived from wheat, maize, and barley was established to be 1000 μg/kg by CODEX Alimentarius [34]. Although the maximum permitted level of NIV has not yet been established, the IC_50_ value of MNV80, which had the highest reactivity, comprises a 28-fold lower concentration than the maximum permitted level of DON. It was thus considered that the sensitivity of the dc-ELISA would be adequate to determine NIV contamination of the aforementioned foods.

### 2.6. Reactivity of PoAbs and MoAbs with 15-Ac-NIV and Other Analogues of NIV

The PoAbs and MoAbs could react with 15-Ac-NIV, which has a similar structure to 15-Glt-NIV with similar or higher reactivity to that with NIV. Since 15-Ac-NIV was not commercially available, it was synthesized and isolated from other products using a procedure similar to that used for 15-Glt-NIV. NIV was reacted with acetic anhydride on ice, and the reaction was stopped before all NIV was acetylated. The product with the same molecular weight as mono-acetylated NIV was isolated and identified as pure 15-Ac-NIV via ^1^H-NMR (Figures S8 and S9).

As expected, the IC_50_ value of the PoAbs with 15-Ac-NIV was 11 ng/mL, which was a 6.4-fold lower concentration than that with NIV (Table 1). The PoAbs reacted with 15-Ac-NIV in the range of 1.7–85 ng/mL as shown in Figure 4a. In contrast, the IC_50_ values of the MoAbs with 15-Ac-NIV were almost the same and were 18 ng/mL, representing a 2-fold lower concentration than that with NIV (Table 1). As shown in Figure 4b,c, the MoAbs, MNV80, and MNV87, reacted with 15-Ac-NIV in the range of 2.8–100 ng/mL and 2.4–95 ng/mL, respectively. These results are easy to understand because the C-15 position of 15-Ac-NIV was esterified with acetic acid instead of the glutaric acid in 15-Glt-NIV, and both ester bonds were electronically neutral and different from the OH group at the C-15 position of NIV, which shows high polarity. However, none of the antibodies reacted with neither 4-acetyl-NIV (4-Ac-NIV or fusarenon X, **E** in Figure 1), DON and its derivatives, 3-acetyl-DON (3-Ac-DON, **F** in Figure 1), nor 15-acetyl-DON (15-Ac-DON; **G** in Figure 1), as shown in Table 1. This result showed that these antibodies could recognize the difference between NIV and DON and the presence or absence of an OH group at the C-4 position. These antibodies also recognized differences between NIV and 4-Ac-NIV. This is because the antibodies raised against 15-Glt-NIV recognize NIV from the opposite side of the C-15 position, such that they recognize substituents on the side of the C-4 position strictly, but recognize those on the side of the C-15 position of NIV loosely at their opening part.

## 3. Conclusions

The antibodies prepared through immunization with the hapten 15-Glt-NIV synthesized in this study showed high specific reactivity with NIV and 15-Ac-NIV. Immunoassays based on these antibodies are thus expected to be effective for detecting NIV, which contaminates grains in the field. A dc-ELISA, which is usually used as an immunoassay for the detection of mycotoxins, was constructed in this study. The applicability of this dc-ELISA will be examined using grain samples in the near future. In addition, the established hapten synthesis method could be useful for the preparation of antibodies against other trichothecene mycotoxins with multiple OH groups. This method is therefore expected to contribute to immunoassays for trichothecene mycotoxins.

## 4. Materials and Methods

### 4.1. Materials

Analytical grade NIV, DON, 4-Ac-NIV, 3-Ac-DON, and 15-Ac-DON purchased from FUJIFILM Wako Pure Chemical Co. (Osaka, Japan) were used as standard reagents. 1-Ethyl-3-(3-dimethylaminopropyl)carbodiimide hydrochloride (EDC) was purchased from Dojindo Molecular Technologies Inc. (Kumamoto, Japan). *N*-hydroxysuccinimide (NHS), KLH, and RPMI 1640 medium were purchased from FUJIFILM Wako Pure Chemical Co. Bovine serum albumin (BSA; Prod. No. A7888), hypoxanthine-aminopterin-thymidine (HAT) medium supplement, and polyethylene glycol (molecular weight 1500) solution were purchased from Sigma-Aldrich Co. (St. Louis, MO, USA). Fetal bovine serum (FBS; HyClone) was purchased from Cytiva (Tokyo, Japan). Ninety-six-well microplates for cell culture (Nunc) and ELISA (Nunc MaxiSorp) and goat anti-mouse IgG (H + L) antibody (Pierce) were purchased from Thermo Fisher Scientific Inc. (Waltham, MA, USA). HRP was purchased from Toyobo Co., Ltd. (Osaka, Japan). Freund’s complete adjuvant (Difco) was purchased from Becton Dickinson and Company (Franklin Lakes, NJ, USA). All other chemicals and reagents used were of analytical grade and purchased from FUJIFILM Wako Pure Chemical Co.

### 4.2. Culture of Fusarium sp. for NIV Production

A strain of NIV-producing *F. kyushuense* [35] was used in this study (MAFF 237646). It was inoculated into potato dextrose agar (Merck, Darmstadt, Germany) and incubated at 20 °C for 1 week. Commercially available edible pressed barley (each batch: 50 g, Kyowaseibaku Co., Kanagawa, Japan) was soaked in an equal weight of tap water in a 500 mL Erlenmeyer flask, incubated overnight at 4 °C, and then autoclaved at 121 °C for 60 min. After cooling, the fungus cultured on potato dextrose agar was cut into 1 cm squares, inoculated into autoclaved barley medium (100 g), and incubated at 20 °C for 4 weeks. The culture medium was used for the extraction of NIV.

### 4.3. Preparation of NIV

The culture medium (three batches: 300 g) was added to 1500 mL of water and incubated for 1 h at 25 °C before mixing using a homogenizer (POLYTRON PT10/35GT Homogenizer, Kinematica AG, Malters, Switzerland) for 5 min. After centrifugation at 1900× *g* for 10 min, the supernatant was added to DIAION HP20 (300 mL; Mitsubishi Chemical, Tokyo, Japan) and incubated for 1 h with stirring. The resin was washed with water (300 mL, 10 times) via decantation before packing it into a glass column (600 mm × 60 i.d. mm). After washing with water (300 mL), NIV was eluted with methanol (450 mL), and the eluate was concentrated in vacuo. These procedures were repeated six times, and 18 batches of the culture medium (1800 g) were used for preparation. The pastes containing NIV were combined and suspended in ethyl acetate/methanol (8:1, *v/v*, 3 mL). Silica gel (1 mL; silica gel 60, particle size 0.063–0.200 mm, Merck) was added to the suspension and dispersed via ultrasonication. The slurry was applied to silica gel (200 mm × 30 i.d. mm; silica gel 60, particle size 0.063–0.200 mm), which was developed using ethyl acetate/methanol (8:1, *v/v*). Fractions containing NIV were collected, concentrated in vacuo, and purified again through silica gel chromatography using the same procedure. The sample purified by performing silica gel chromatography was dissolved in 1 mL of 5% (*v/v*) acetonitrile in water and placed in a refrigerator (4 °C). A white powder comprising NIV (200 mg) was obtained as the precipitate.

### 4.4. HPLC Analysis of NIV

The amount of NIV in the fractions during the purification procedure was analyzed using a reversed-phase HPLC system equipped with UV detection under the following conditions: system, Agilent 1100 series (Agilent Technologies, Santa Clara, CA, USA); column, TSKgel ODS-80Ts (250 mm × 4.6 i.d. mm, 5 μm, Tosoh, Tokyo); eluent, water/acetonitrile (95:5, *v/v*); flow rate, 1 mL/min; detection wavelength, 220 nm; oven temperature, 40 °C. Under these conditions, NIV was detected at a retention time of approximately 10 min. Before HPLC analysis, the aqueous extract of the culture medium was pretreated using the following procedure: The extract was diluted 3/20 with acetonitrile, 10 mL of which was loaded onto a cartridge (Multisep #227 Trich+, Romer Labs, Gatzersdorf, Austria). The eluate (4 mL) was collected after excluding the first eluate (3 mL) and was concentrated in vacuo.

### 4.5. Synthesis of 15-Glt-NIV and 15-Ac-NIV

15-Glt-NIV (**D** in Figure 1) was prepared based on a moderate reaction between purified NIV and glutaric anhydride. This reaction was monitored by the HPLC-UVD under the following conditions: pump, LC-10A (Shimadzu, Kyoto, Japan); column, YMC-Pack ODS-A (150 mm × 4.6 i.d. mm, 5 μm, YMC, Kyoto, Japan); eluent, solution A (0.1% (*v/v*) trifluoroacetic acid in water) and solution B (0.1% (*v/v*) trifluoroacetic acid in acetonitrile), 1–60% (*v/v*) B for 0–25 min with a linear gradient; flow rate, 1 mL/min; detector, SPD-M10A (Shimadzu); detection wavelength, 220 nm; oven temperature, 40 °C and by the LC-MS under the following conditions: system, Agilent 1260 infinity II HPLC system (Agilent Technologies); column, YMC-Pack ODS-A (150 mm × 4.6 i.d. mm, 5 μm, YMC); eluent, solution A (0.05% (*v/v*) trifluoroacetic acid in water) and solution B (0.05% (*v/v*) trifluoroacetic acid in acetonitrile), 1–60% (*v/v*) B for 0–25 min with a linear gradient; flow rate, 1 mL/min; oven temperature, 40 °C; ionization, electrospray ionization (ESI positive and negative).

NIV (20 mg), glutaric anhydride (23 mg), and 4-dimethylaminopyridine (2.4 mg) were dissolved in a mixture of 1 mL of dimethyl sulfoxide and 10 mL of chloroform and left for 3 h at 25 °C with stirring. After extraction with 1% (*v/v*) acetic acid in water, the water layer was subjected to preparative HPLC under the following conditions: pump, LC-8A (Shimadzu, Kyoto, Japan); column, YMC-Pack ODS-A (250 mm × 30 i.d. mm, 5 μm, YMC, Kyoto, Japan); eluent, solution A (0.05% (*v/v*) trifluoroacetic acid in water) and solution B (0.05% (*v/v*) trifluoroacetic acid in acetonitrile), 0–40% (*v/v*) B for 0–100 min with a linear gradient; flow rate, 20 mL/min; detector, SPD-10AV (Shimadzu); and detection wavelength, 220 nm. The eluate containing 15-Glt-NIV was then collected and freeze-dried. A white powder comprising 15-Glt-NIV (2 mg) was obtained. The unreacted NIV was recovered and reused in the reaction. 15-Glt-NIV (22 mg) was obtained from 110 mg of NIV by repeating this procedure. Spectroscopic measurements of 15-Glt-NIV were conducted using LC-MS (Agilent G1956B LC/MSD detector with Agilent 1260 infinity II HPLC system, Agilent Technologies) and NMR (JEOL ECX400 spectrometer, JEOL, Tokyo). The spectral data (Figures S2, S5 and S10) are as follows: ESI-MS (*m/z*) 425.1 [M − H]^−^, calcd. for C_20_H_25_O_10_ 425.1; 449.1 [M + Na]^+^, calcd. for C_20_H_26_O_10_Na 449.1; ^1^H NMR (CDCl_3_, 400 MHz) δ (ppm), 6.66 (qd, 1H, *J* = 1.8, 6.0 Hz), 4.90 (s, 1H), 4.67 (d, 1H, *J* = 6.0 Hz), 4.53 (d, 1H, *J* = 3.2 Hz), 4.42 (d, 1H, *J* = 12.8 Hz), 4.34 (dd, 1H, *J* = 3.2, 4.6 Hz), 4.07 (d, 1H, *J* = 12.4 Hz), 3.80 (d, 1H, *J* = 5.0 Hz), 3.09 (d, 1H, *J* = 4.1 Hz), 3.06 (d, 1H, *J* = 4.1 Hz), 2.41 (t, 2H, *J* = 6.9 Hz), 2.35 (td, 1H, *J* = 6.9, 16.0 Hz), 2.30 (td, 1H, *J* = 6.9, 16.0 Hz), 1.99–1.88 (m, 5H), 1.11 (s, 3H).

15-Ac-NIV (**C** in Figure 1) was prepared based on the following reaction between purified NIV and acetic anhydride: NIV (5 mg) was dissolved in 500 μL of pyridine under shading conditions. After adding ice cold acetic anhydride (2.5 mL) to the solution, the mixture was left on ice for 10 min. An aliquot (45 mL) of methanol/water (25:75 *v/v*) was added to the reaction mixture to stop the reaction. The solution was concentrated in vacuo to approximately 1 mL and was subjected to preparative HPLC under the following conditions: system, Agilent 1100 series (Agilent Technologies); column, Inertsil ODS-3 (250 mm × 10 i.d. mm, 5 μm, GL Science, Tokyo, Japan); eluent, methanol/water (1:3 *v/v*); flow rate, 2.75 mL/min; detection wavelength, 220 nm. The eluate containing 15-Ac-NIV (retention time of 28–30 min) was collected and evaporated. White 15-Ac-NIV powder (4 mg) was obtained. Spectroscopic measurements of 15-Ac-NIV were conducted using the following instruments: high resolution LC-MS (LC-HRMS; Orbitrap MS “Exactive,” Thermo Fisher Scientific, Waltham, MA, USA) and NMR (Bruker Avance III 600 MHz, Bruker Biospin, Billerica, MA, USA). The spectral data (Figures S8 and S9) were as follows: ESI-MS (*m/z*) 413.1460 [M + CH_3_COO]^−^, calculated. for C_19_H_25_O_10_ 413.1442; ^1^H NMR (CD_3_OD, 600 MHz) δ (ppm), 6.59 (qd, 1H, *J* = 1.5, 5.9 Hz), 4.87 (s, 1H), 4.60 (d, 1H, *J* = 5.9 Hz), 4.37 (d, 1H, *J* = 12.3 Hz), 4.33 (d, 1H, *J* = 3.4 Hz), 4.25 (d, 1H, *J* = 12.2 Hz), 4.13 (dd, 1H, *J* = 3.5, 4.7 Hz), 3.59 (d, 1H, *J* = 4.7 Hz), 2.97 (dd, 2H, *J* = 4.4, 13.9 Hz), 1.89 (s, 3H), 1.84 (s, 3H), 1.05 (s, 3H).

### 4.6. Preparation of 15-Glt-NIV–Protein Conjugate

The carboxy group of the synthesized 15-Glt-NIV was covalently bound to primary amines in lysine residues of KLH or HRP via the active ester method, as described previously [36]. The NIV-KLH conjugate was used as an immunogen, and HRP-labeled NIV was used as the labeled antigen for d-ELISA and dc-ELISA. Briefly, 100 μL of 15-Glt-NIV (5 μmol) was dissolved in anhydrous dimethyl sulfoxide and mixed with 5 μL of NHS (6 μmol) and 10 μL of EDC (6 μmol) dissolved in anhydrous dimethyl sulfoxide. The mixture was left at room temperature for 1.5 h. Meanwhile, each protein sample (10 mg) was dissolved in 1 mL of phosphate buffered saline (PBS; 10 mmol/L phosphate buffer and 150 mmol/L NaCl; pH 7.0) and added to the mixture. The mixture was left at room temperature for 1.5 h to complete the reaction. The NIV-KLH conjugate was purified via dialysis against PBS at 4 °C for 3 days through the exchange of PBS three times. Meanwhile, the HRP-labeled NIV was purified via gel filtration chromatography (Sephadex G-25, superfine grade, Cytiva) using an open column (250 mm ×10 i.d. mm).

### 4.7. Preparation of PoAbs and MoAbs

PoAbs and MoAbs were prepared as described previously [36]. Briefly, BALB/c mice (7-week-old females) were obtained from Japan SLC, Inc. (Shizuoka, Japan). Three mice were intraperitoneally immunized with 100 μL of NIV-KLH conjugate (100 μg/mouse) in Freund’s complete adjuvant. For a 1-month interval, mice were further intraperitoneally immunized with 100 μL of the NIV-KLH conjugate (25 μg/mouse) in Freund’s incomplete adjuvant. Blood samples of 50 μL were collected from the tail vein of each mouse 1 week after each immunization. Serum prepared from blood was used as the PoAbs without purification.

Meanwhile, 3 days after the last immunization, the spleen was removed from the immunized mouse, and spleen cells were collected. The cells were fused with P3U1 myeloma cells at a ratio of 5:1 using a polyethylene glycol solution. The fused cells were suspended at a concentration of 2 × 10^5^ cells/mL in HAT medium with RPMI 1640 medium, 10% FBS, and HAT medium supplements and transferred to 96-wells microplates. After incubation at 37 °C for 7–10 days, colonies that formed and secreted anti-NIV MoAbs were assessed using d-ELISA and dc-ELISA. Selected hybridoma cells were cloned using the limiting dilution method. The culture supernatant of the cloned cells was used as the MoAbs.

### 4.8. d-ELISA and dc-ELISA Creation

d-ELISA was performed as described previously [36]. One hundred microliters of anti-mouse IgG antibody (goat, 4 μg/mL) dissolved in PBS was added to each well of a 96-well microtiter plate and incubated at 4 °C overnight. After removing the solution, 300 μL of 0.4% BSA dissolved in PBS was added to the wells and incubated at 25 °C for 1 h. After washing once with PBS supplemented with 0.02% Tween 20, 100 μL of serially diluted PoAbs or MoAbs with PBS containing 0.2% BSA was added to the wells and incubated at 25 °C for 1 h. After washing three times, 100 μL of HRP-labeled NIV (50 ng/mL) in PBS containing 0.2% BSA was added to the wells and incubated at 25 °C for 1 h. The wells were washed three times, and 100 μL of the color development solution (100 μg/mL 3,3′,5,5′-tetramethylbenzidine, 0.006% H_2_O_2_, and 0.1 mol/L acetate buffer; pH 5.5) was added to the wells. After 10 min, 100 μL of 0.5 mol/L sulfuric acid was added to stop the color development reaction. Absorbance was measured at 450 nm using an xMark microplate reader (Bio-Rad Laboratories, Hercules, CA, USA).

The dc-ELISA was generated based on d-ELISA [36]. The anti-mouse IgG antibody (goat) and 0.4% BSA in PBS were used as well as to the d-ELISA. After washing once, the PoAbs (30,000-fold dilutions for antiserum after the 1st booster immunization and 100,000-fold dilutions for antiserum after the 2nd and 3rd booster immunizations) or MoAbs (50-fold dilutions for MNV80 and 100-fold dilutions for MNV87), of which the dilution rates were adjusted to the absorbance 1–1.5 for the above d-ELISA, were added to the wells and incubated at 25 °C for 1 h. HRP-labeled NIV (100 ng/mL) was mixed with an equal volume of each solution of NIV or its related mycotoxins (0.5–1000 ng/mL) in 10% methanol in water. After three washes, the mixture was added to the wells and incubated at 25 °C for 1 h. Further, the wells were washed thrice and treated with the color development solution and sulfuric acid, and absorbance was measured at 450 nm using d-ELISA.

## 5. Patents

Azabu University has a patent pending for the preparation methods of monoclonal antibodies targeting NIV.

## Figures and Tables

**Table 1:** Fifty percent inhibitory concentration (IC_50_) values of each antibody with nivalenol (NIV) and its derivatives via direct competitive enzyme-linked immunosorbent assay.

NIV and Its Derivatives	PoAb	MoAb	
		MNV80	MNV87
NIV	70 *	36	37
4-Ac-NIV	>1000	>1000	>1000
15-Ac-NIV	11	18	18
DON	>1000	>1000	>1000
3-Ac-DON	NT	>1000	>1000
15-Ac-DON	NT	>1000	>1000

* IC_50_ values (ng/mL). Each data point is the mean of three replicates based on independent examinations.

## Data Availability

Not applicable.

## Supplementary Materials

The following supporting information can be downloaded at: https://www.mdpi.com/article/10.3390/toxins14110747/s1, Figure S1: Representative chromatogram from obtained nivalenol (NIV) based on high-performance liquid chromatography equipped with a UV detector. Figure S2: Mass spectrum of the peak at the retention time of 14.4 min in the chromatogram of Figure 2; Figure S3: Mass spectrum of the peak at the retention time of 14.7 min in the chromatogram of Figure 2; Figure S4: Mass spectrum of the peak at the retention time of 18.0 min in the chromatogram of Figure 2; Figure S5: ^1^H-NMR spectrum of the purified compound from the peak at the retention time of 14.4 min in the chromatogram of Figure 2; Figure S6: ^1^H-NMR spectrum of the purified compound from the peak at the retention time of 14.7 min in the chromatogram of Figure 2; Figure S7: ^1^H-NMR spectrum of the purified compound from the peak at the retention time of 18.0 min in the chromatogram of Figure 2; Figure S8: Mass spectrum of purified 15-acetylnivalenol; Figure S9: ^1^H-NMR spectrum of purified 15-acetylnivalenol; Figure S10: Mass spectrum of 15-glutarylnivalenol.
