# Supplementary material for: Preparation of Monoclonal Antibodies Specifically Reacting with the Trichothecene Mycotoxins Nivalenol and 15-Acetylnivalenol via the Introduction of a Linker Molecule into Its C-15 Position
Source: Toxins (Basel). 2022 Oct 31;14(11):747. doi: 10.3390/toxins14110747 (PMC9693464; doi:10.3390/toxins14110747)
Supplement: Supplementary file 1 [file toxins-14-00747-s001.zip › toxins-1958977-SI.pdf]

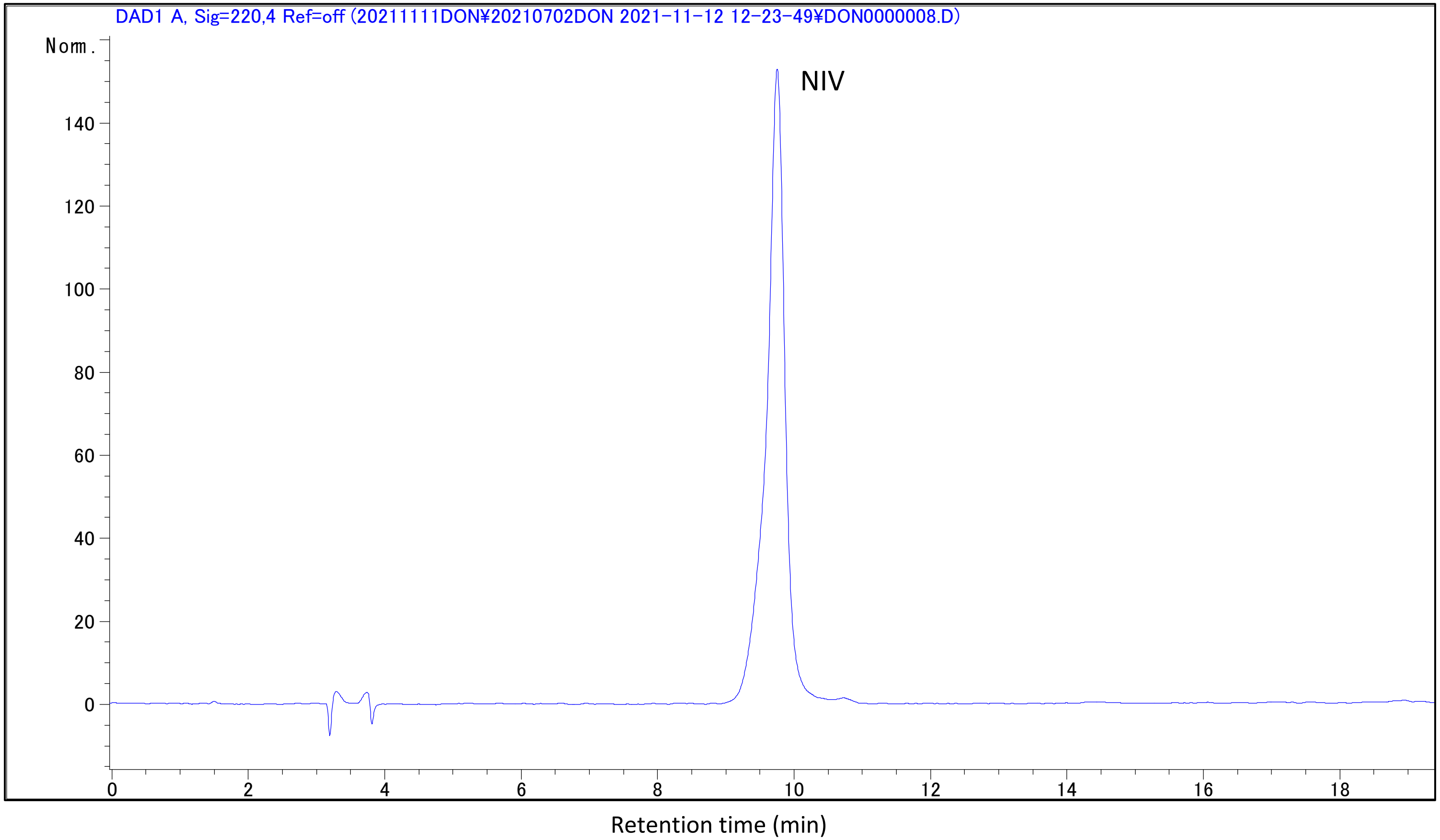

Figure S1. Representative chromatogram from obtained nivalenol (NIV) based on high-performance liquid chromatography equipped with a UV detector.

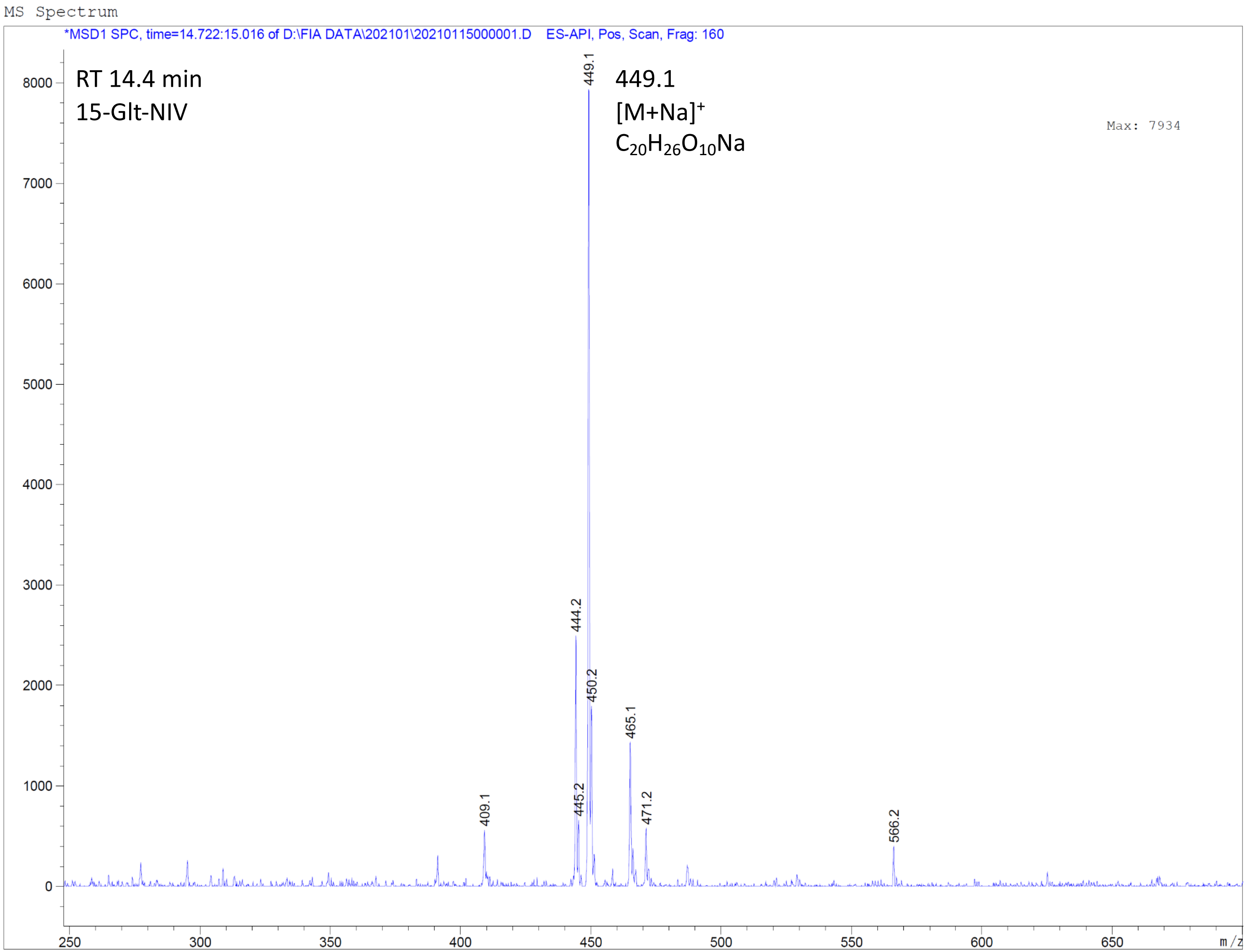

Figure S2. Mass spectrum of the peak at the retention time of 14.4 min in the chromatogram of Figure 2.

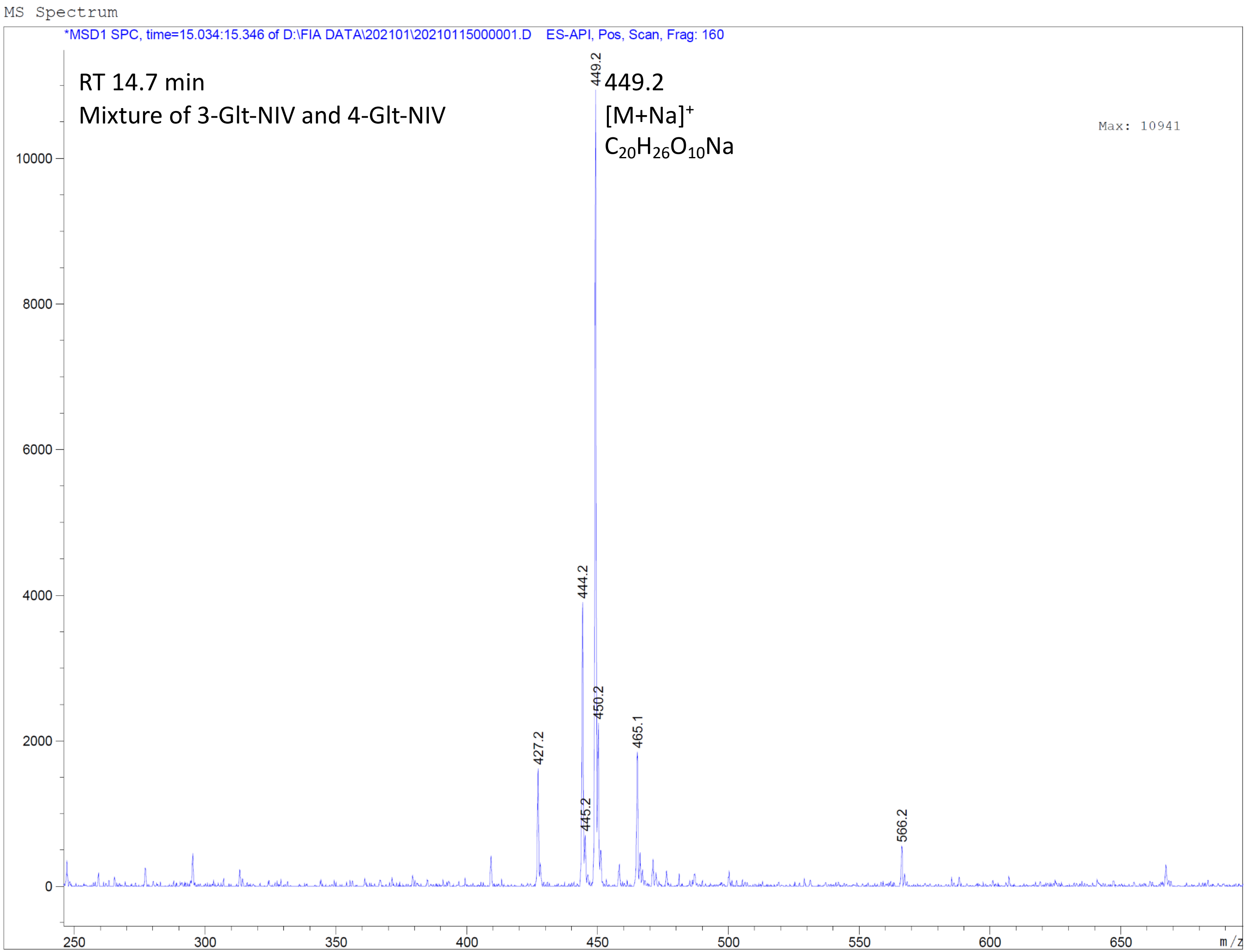

Figure S3. Mass spectrum of the peak at the retention time of 14.7 min in the chromatogram of Figure 2.

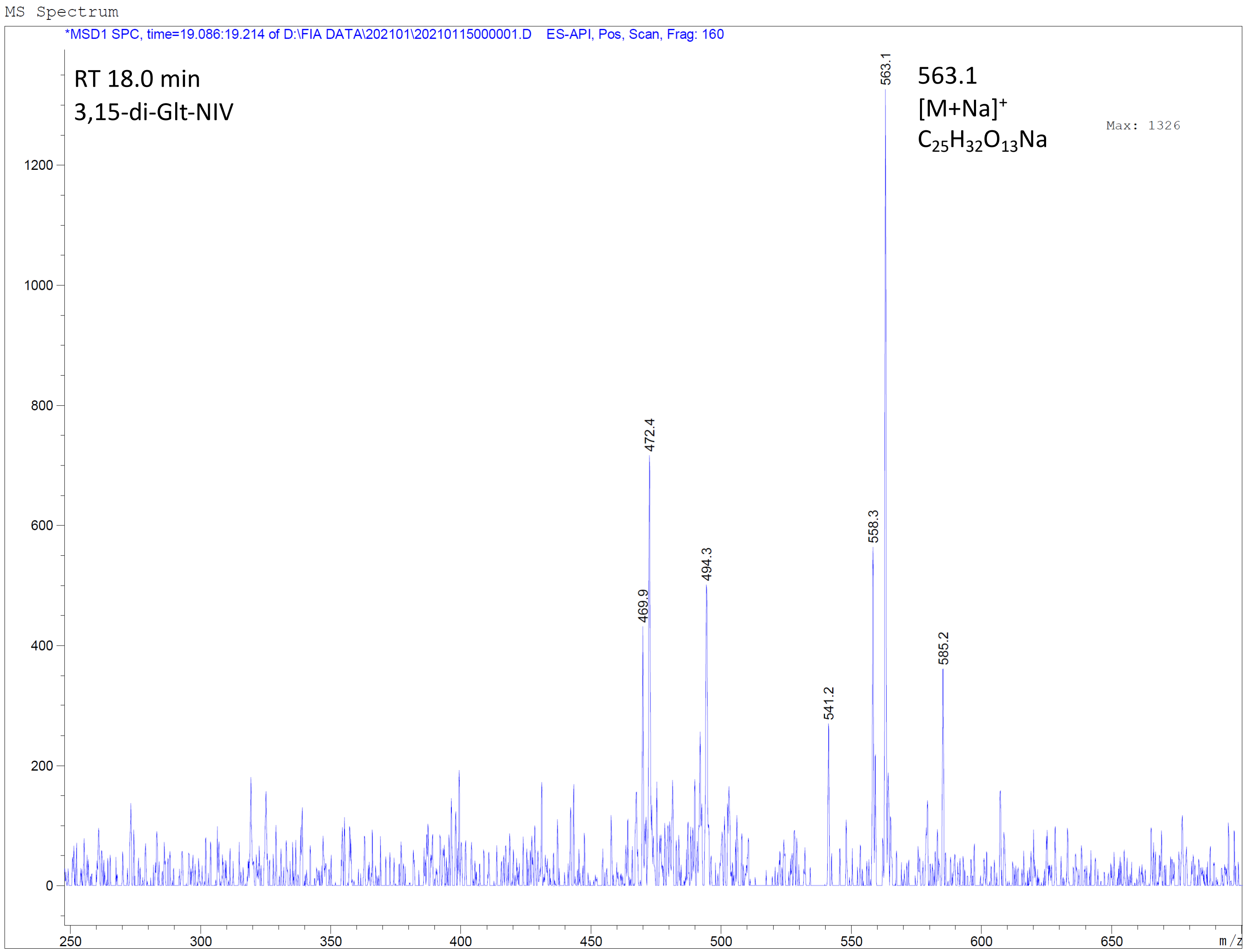

Figure S4. Mass spectrum of the peak at the retention time of 18.0 min in the chromatogram of Figure 2.

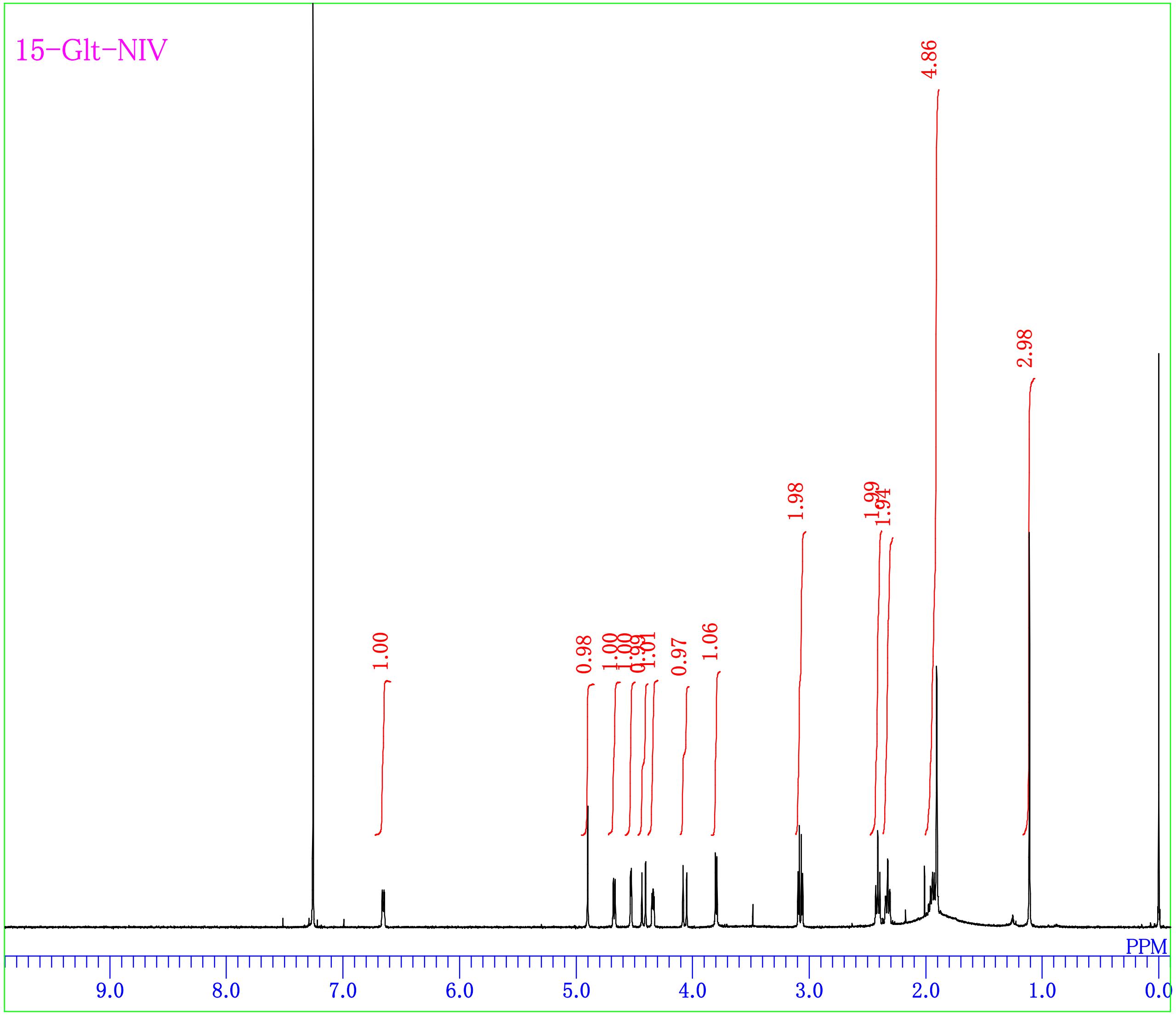

Figure S5. ^{1}H-NMR spectrum of the purified compound from the peak at the retention time of 14.4 min in the chromatogram of Figure 2.

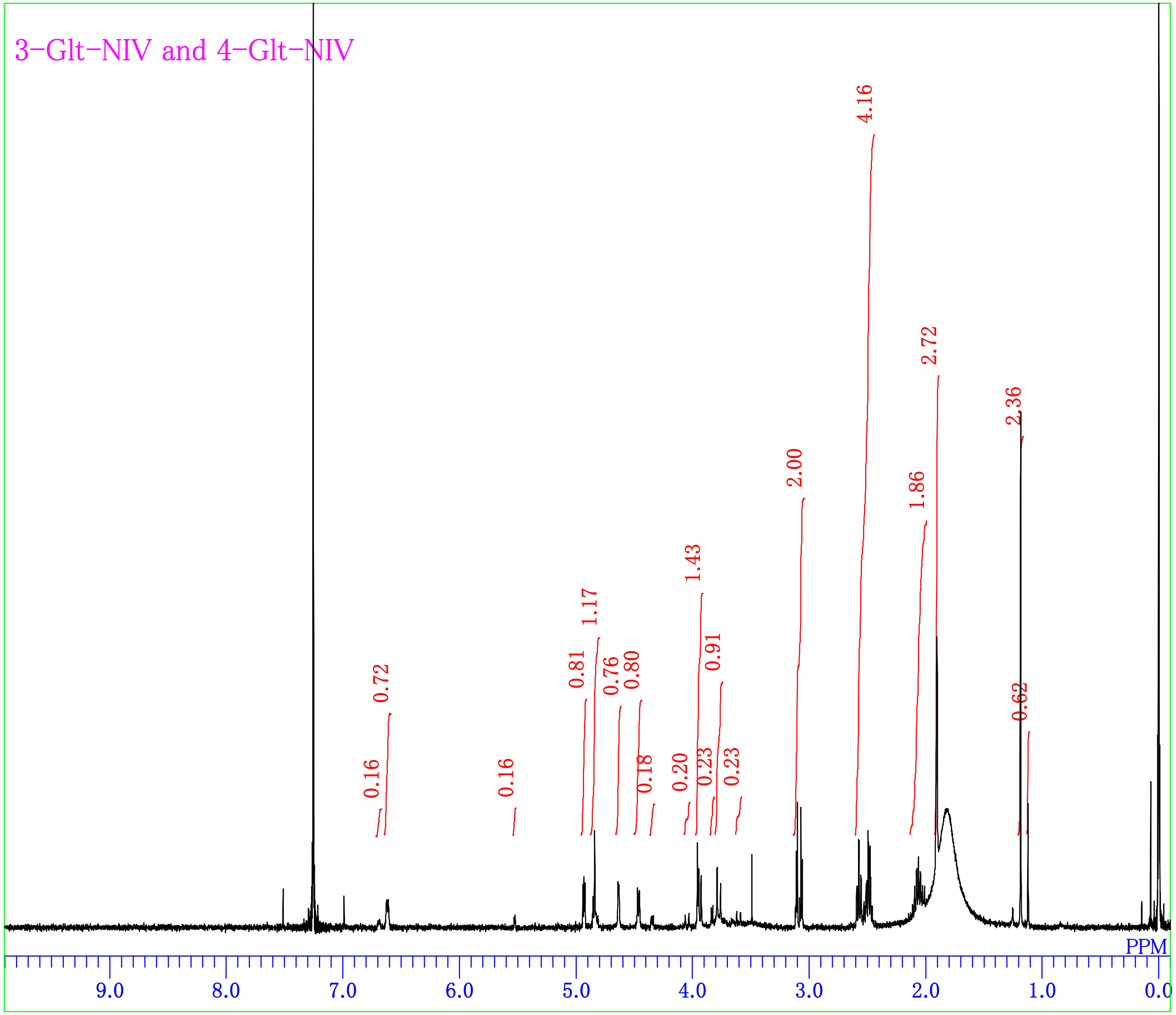

Figure S6. ^{1}H-NMR spectrum of the purified compound from the peak at the retention time of 14.7 min in the chromatogram of Figure 2 .

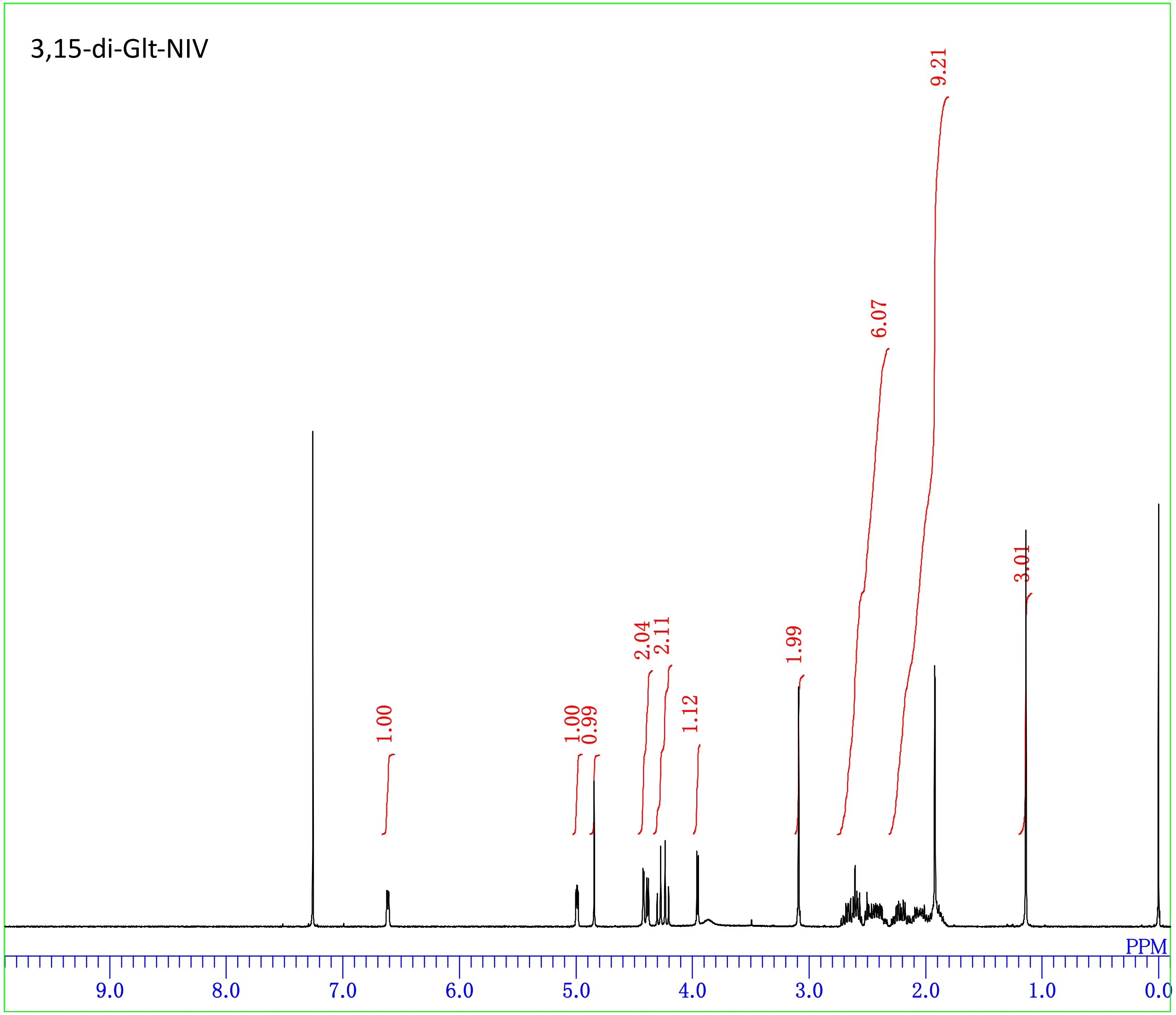

Figure S7. ^{1}H-NMR spectrum of the purified compound from the peak at the retention time of 18.0 min in the chromatogram of Figure 2.

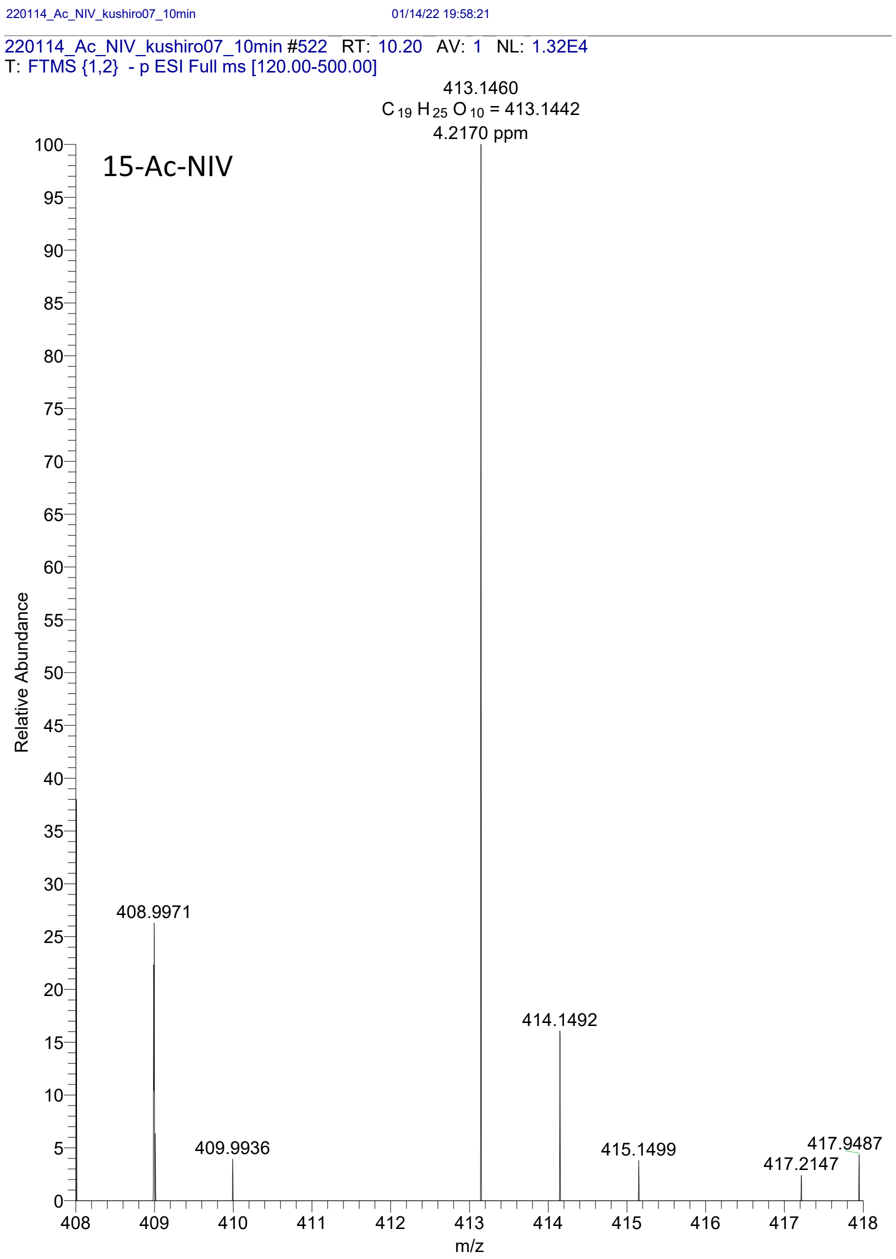

Figure S8. Mass spectrum of purified 15-acetylnivalenol.

15-Ac-NIV

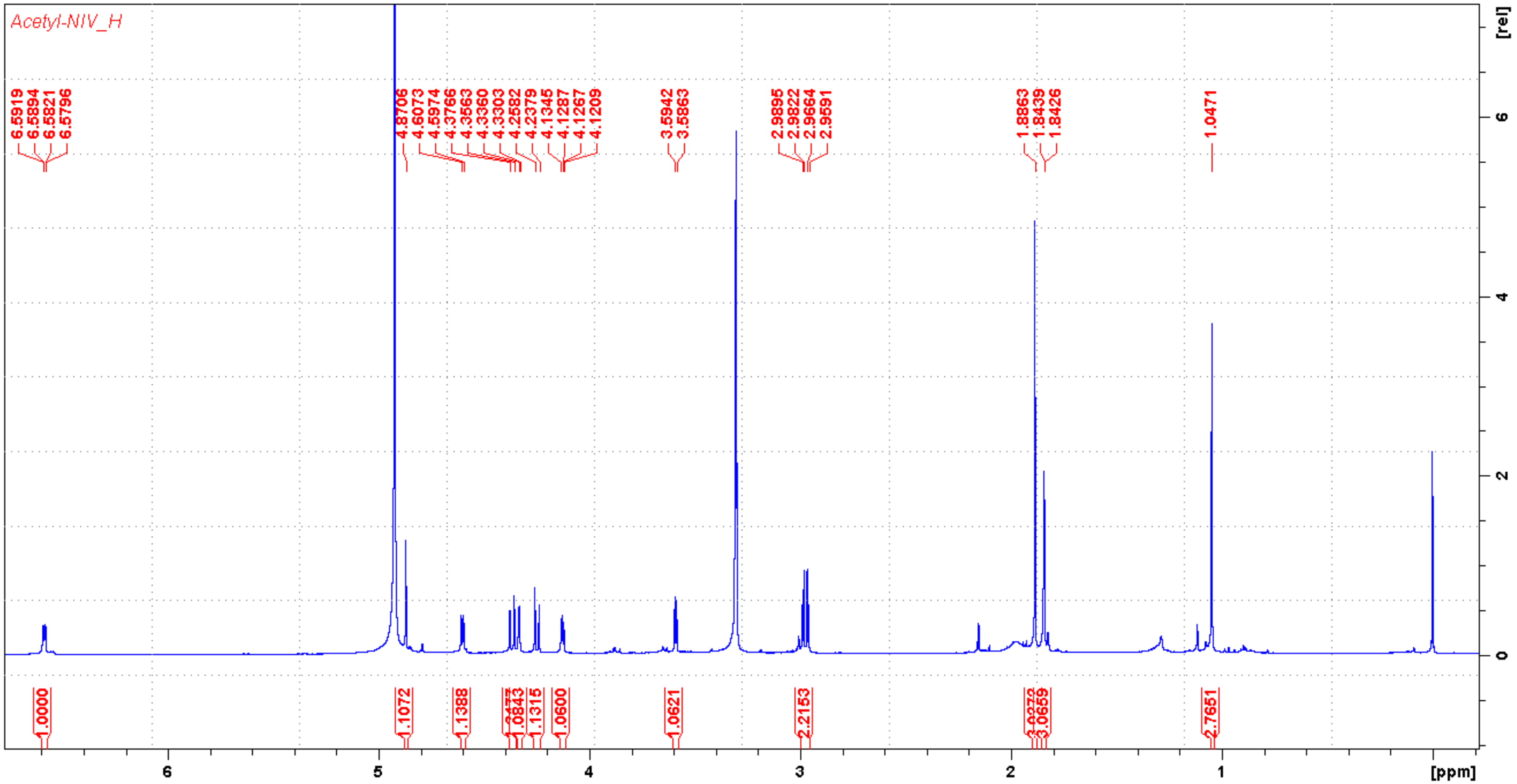

Figure S9. ^{1}H-NMR spectrum of purified 15-acetylnivalenol.

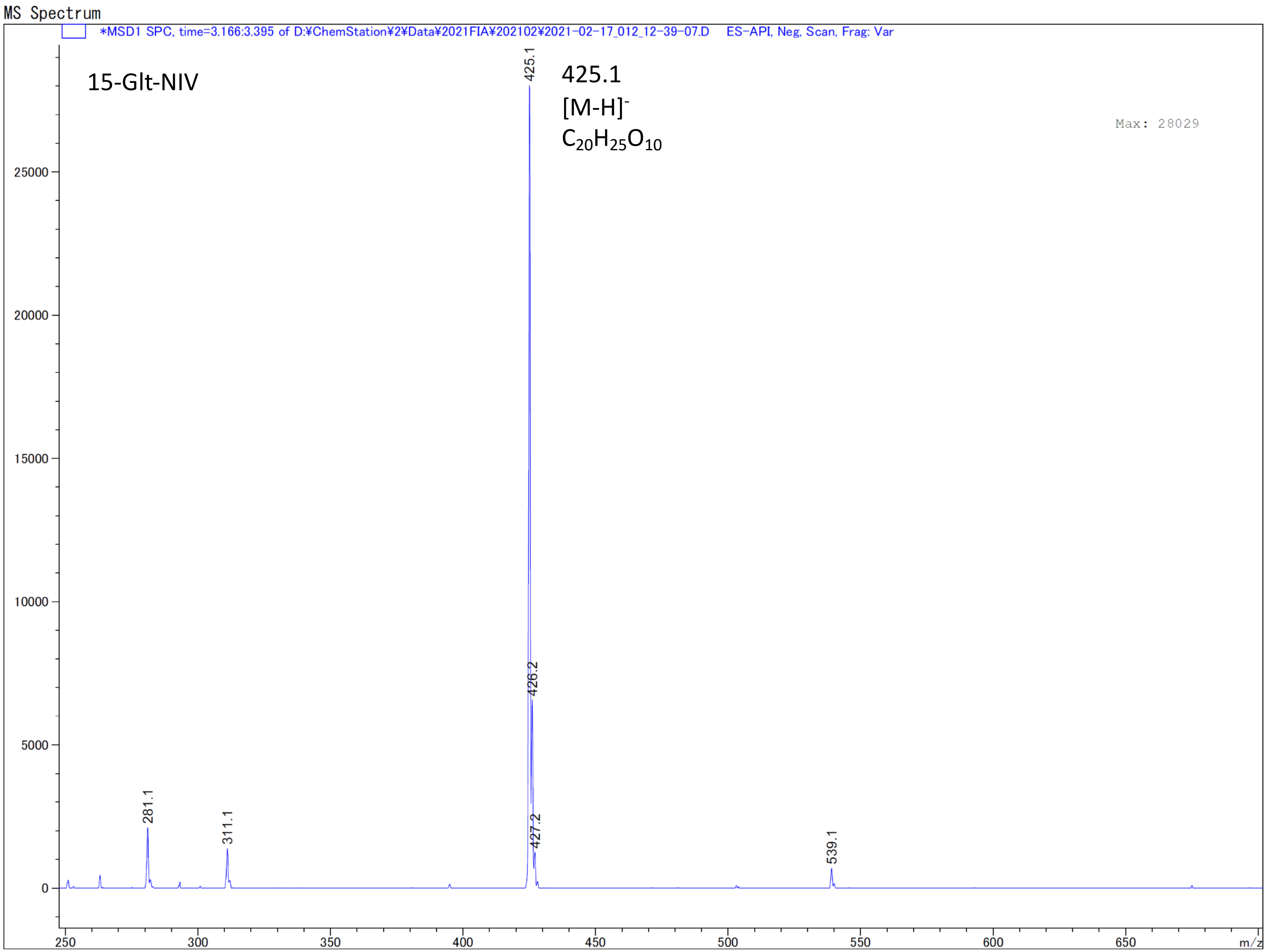

Figure S10. Mass spectrum of 15-glutarylnivalenol.
